# Brain Drain and Retention Strategies: Lived Experience of Expatriate Nurses in Saudi Arabia: Challenges and Implications

**DOI:** 10.1155/jonm/9947313

**Published:** 2025-08-06

**Authors:** Ebtsam Aly Abou Hashish, Hend Abdou Alnajjar

**Affiliations:** ^1^College of Nursing – Jeddah, King Saud bin Abdul-Aziz University for Health Sciences, Jeddah, Saudi Arabia; ^2^King Abdullah International Medical Research Center, Jeddah, Saudi Arabia; ^3^Faculty of Nursing, Alexandria University, Alexandria, Egypt

**Keywords:** brain drain, expatriate nurses, lived experience, migration, nurses, nursing retention strategies, qualitative design, Saudi Arabia, Saudi healthcare system

## Abstract

**Aim:** This study explored the lived experiences of expatriate nurses in Saudi Arabia, examining the factors driving migration and brain drain and identifying retention strategies from their perspectives.

**Background:** The brain drain phenomenon, marked by the migration of skilled nurses from developing to wealthier countries for better career opportunities and living conditions, remains a global healthcare concern. Despite growing attention, research on factors driving expatriate nurses' migration to Saudi Arabia is limited. Understanding these factors is crucial for developing effective retention strategies.

**Methods:** A phenomenological qualitative approach was used with a purposive sample of 36 expatriate nurses from diverse nationalities such as Filipino, Indian, South African, Malaysian, Jordanian, and the United Kingdom. Data were collected through semistructured interviews and analyzed thematically.

**Findings:** Four themes were identified shaping the brain drain phenomenon among expatriate nurses in Saudi Arabia: push factors, pull factors, challenges, and retention strategies. Within these, 15 subthemes and 31 related factors emerged. Economic hardship, limited career growth, and poor working conditions drove migration, while higher salaries, career advancement, and better work environments attracted nurses. Challenges included high patient loads, restricted leadership roles, social adaptation difficulties, and work–life imbalance. Most expatriate nurses (86.1%, *n* = 31) intended to remain in Saudi Arabia.

**Conclusion:** Findings underscore the complicated connection between push and pull factors and challenges influencing expatriate nurse's migration and retention. This study contributes new insights into nursing workforce management in Saudi Arabia and offers policy-driven recommendations to enhance nurse retention strategies, supporting the Saudi Vision 2030 healthcare transformation.

**Implications for Nursing Management:** Addressing these challenges requires a multifaceted approach that includes financial incentives, structured career development programs, leadership inclusion, work–-life balance policies, and expatriate support systems.

## 1. Background

Nursing is a high-demand profession that requires extensive effort to maintain continuity of care in hospitals [1]. The increasing workload and pressures on nurses, particularly in underresourced healthcare systems, often lead them to think of migration [2, 3]. The phenomenon of brain drain, also referred to as human capital flight or migration, refers to the migration of highly educated and skilled professionals, such as nurses, from developing countries to more affluent nations in search of better wages, access to advanced technology, and improved sociopolitical conditions [4, 5]. This trend has created significant workforce imbalances, affecting healthcare service delivery and patient outcomes in both source and destination countries [6].

Global migration and healthcare workforce mobility have become increasingly complex, as healthcare systems struggle to manage the movement of professionals. Migrant healthcare workers, particularly nurses, play a crucial role in responding to global health crises and ensuring universal health coverage [7]. The migration and retention of nurses present substantial challenges, particularly in the Middle Eastern healthcare systems, where they significantly impact the size and quality of the nursing workforce, ultimately influencing care standards [5]. A deeper exploration of these issues is vital to addressing workforce stability and improving healthcare service provision.

The pattern of relying on international healthcare professionals mirrors the practices observed in certain developed nations that similarly recruit from abroad to meet their staffing requirements. Saudi Arabia has emerged as a significant hub for migrant healthcare professionals [8]. The Ministry of Health (MOH), in collaboration with the military and private healthcare sectors, oversees the healthcare system in Saudi Arabia. According to a 2023 MOH report, the total number of nurses reached over 235,461 across public and private sectors [9]. Of these, approximately 60%–70% are expatriate or non-Saudi nurses, with the majority originating from India, the Philippines, and Malaysia [10, 11]. Despite increased efforts to train and integrate Saudi nationals into the healthcare workforce over the past 2 decades, the country continues to rely on migrant healthcare professionals to meet its healthcare demands, particularly in specialized fields [12]. A deeper exploration of factors affecting expatriate retention is vital to addressing workforce stability and improving healthcare service provision.

### 1.1. Conceptual Framework

**Figure 1:**
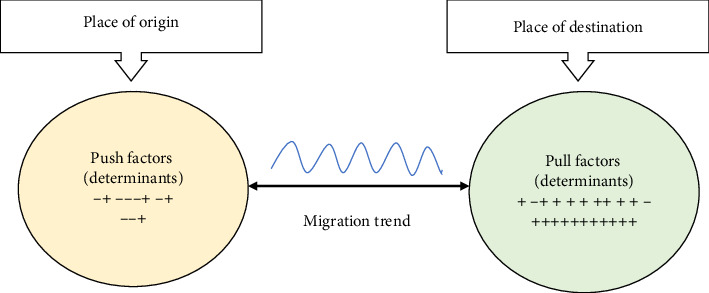
Migration theory's push and pull factors [3].

The migration of nurses is frequently analyzed through the push–pull framework, which examines the key factors influencing migration decisions. This study aligns with Lee's migration theory [13]. This highlights the connection between push factors in the home country and pull factors in the destination country, illustrating a reciprocal relationship between these two factors. (see Figure 1).

Push factors are internal conditions in the source country that drive nurses to migrate [5]. These include economic hardships, social challenges, political instability, poor healthcare systems, professional limitations, job insecurity, and workplace violence, all of which contribute to nurses seeking employment opportunities abroad [14, 15]. Conversely, pull factors are incentives in host countries that attract nurses, including better living standards, fair governance, and educational opportunities. Professional growth, job stability, and access to advanced technology enhance career prospects, while supportive work environments, adequate staffing, and work–life balance improve job satisfaction [3, 8, 16]. Competitive salaries, comprehensive benefits, and strong organizational support further influence migration decisions among nurses [2, 8]. One key reason for Saudi Arabia's continued reliance on expatriate nurses is the persistent difficulty in recruiting and educating sufficient numbers of Saudi nationals to enter and complete nursing education programs [11, 17].

Numerous studies have explored the nurse brain drain phenomenon in developing nations, identifying a range of contributing factors. For instance, in Africa, Pretorius [14] reported that low salaries, which fail to meet nurses' basic needs and responsibilities, are a major push factor. Similarly, Okafor and Chimereze [18] found that poor remuneration, unfavorable working conditions, and weak governmental policies motivate Nigerian nurses to migrate. These challenges are often compounded by pull factors such as higher salaries, better professional opportunities, and improved healthcare systems in destination countries. Buchan et al. [19] further emphasized the ongoing workforce crisis caused by aggressive international recruitment, which continues to deplete skilled healthcare professionals from African countries.

In Asia, Thapa and Shrestha [20] identified low wages and poor working conditions as key drivers of nurse migration from Nepal, while Kadel and Bhandari [4] highlighted dissatisfaction with salaries and limited career growth as significant push factors. Bourgeault et al. [15], through a multimethod study in the Philippines, India, and South Africa, underlined that inadequate remuneration remains a major driver of nurse migration. In addition, Konlan et al. [2] synthesized evidence from lower- and middle-income countries (LMICs), identifying poor salaries, challenging working conditions, outdated healthcare technologies, limited job opportunities, insecurity, high crime rates, political corruption, and language barriers as key migration determinants.

Within the Mediterranean region, Abou Hashish and Ashour [5] conducted a mixed-methods study investigating nurse brain drain, and later, Abou Hashish et al. [3] developed the Brain Drain Questionnaire (BDQ) to examine the determinants influencing nurse migration both domestically and internationally. In Saudi Arabia, Almansour et al. [8] explored factors attracting nurses and doctors, highlighting the pull factors influencing expatriate recruitment. In addition, in Europe, research by Mara [21] and Jurić [22] examined nurse migration, providing further insights into workforce mobility trends.

Nursing turnover is a key driver of brain drain, contributing to global imbalances in healthcare workforce distribution and undermining service quality, access, and system resilience. In Saudi Arabia, the high turnover of expatriate nurses reflects broader migration trends and poses ongoing challenges to workforce stability [23]. Aljohani and Alomari [24] reported that 18.3% of Filipino nurses identified low salaries and heavy workloads as primary reasons for leaving their jobs, factors commonly associated with international nurse migration, although the sample was not fully representative. Similarly, Kaddourah et al. [25] found that the vast majority of nurses in their study (97% expatriates) expressed dissatisfaction with their work environment, which was strongly associated with turnover intention and the likelihood of seeking employment elsewhere.

These trends mirror the structural pressures that drive skilled nurses to migrate, contributing to a persistent shortage in the local workforce. Alluhidan et al. [17] emphasized that early career exits and insufficient entry of new nurses into the profession have intensified the national shortage in Saudi Arabia, a situation made more complex by rapid population growth, shifting health needs, and accelerated healthcare system expansion [26]. Al-Hanawi et al. [27] further noted that rising life expectancy, from 75 years in 2015 to a projected 80 by 2030, has increased the demand for trained healthcare personnel. At the same time, Alsadaan et al. [11] highlighted the ongoing difficulties in attracting Saudi nationals to nursing education, reinforcing the country's reliance on expatriate nurses and its vulnerability to the effects of international brain drain. These findings underscore the need for targeted, context-sensitive retention strategies that address the migration pressures and lived experiences of expatriate nurses working in Saudi Arabia.

### 1.2. Significance of the Study

While previous studies have examined nurse migration, they focus largely on premigration motivations rather than postmigration challenges and long-term retention. This leaves a gap in understanding expatriate nurses' experiences, particularly in Saudi Arabia, where reliance on foreign healthcare workers is significant. Despite substantial investments in workforce recruitment, high turnover among expatriate nurses persists, leading to workforce instability, disruptions in patient care, and challenges in service quality [8, 28]. Qualitative research on migration factors from the direct perspective of expatriate nurses in Saudi Arabia remains limited. Understanding why some nurses stay while others leave is essential for developing evidence-based retention strategies tailored to their needs.

Therefore, this study addresses the research gap by examining the lived experiences of expatriate nurses in Saudi Arabia, identifying challenges and barriers to retention that enhance workforce stability. It explores premigration motivations and postmigration challenges to provide a comprehensive understanding of career sustainability. Findings will inform nursing workforce policies, improve workplace conditions, support career advancement, and enhance expatriate support systems. Aligning with Saudi Arabia's Vision 2030, the study contributes to workforce sustainability, service quality, and regional and international migration policies, offering evidence-based recommendations for stabilizing the nursing workforce in Saudi Arabia and beyond.

### 1.3. Aim of the Study

This study explored the lived experiences of expatriate nurses in Saudi Arabia, examining the factors driving migration and brain drain and identifying retention strategies from their perspectives.

## 2. Methods

### 2.1. Study Design and Setting

This study adopted a qualitative phenomenological approach to explore the lived experiences of expatriate nurses working in Saudi Arabia. Phenomenology was deemed appropriate as it facilitates a deep understanding of individuals' subjective perspectives, particularly regarding their motivations for migration and decisions related to retention within the Saudi healthcare context [29]. To ensure methodological rigor, the study adhered to the Consolidated Criteria for Reporting Qualitative Research (COREQ) guidelines and followed the COREQ checklist in reporting its findings (Supporting File). Data were collected at a prominent tertiary care hospital located in the western region of Saudi Arabia. This hospital, comprising 750 beds and accredited by the Joint Commission International (JCI), serves as a major referral center, known for its multicultural nursing workforce and wide range of specialized healthcare services.

### 2.2. Study Subjects and Sampling

The study population consisted of 36 employed expatriate (migrant) nurses working at the selected hospital. Eligible participants were nurses with a minimum of 6 months of work experience in Saudi Arabia and available during the data collection period. Saudi national nurses were excluded to ensure the focus remained on the experiences of foreign nurses. A purposive sampling strategy was applied to recruit participants until data saturation was achieved, meaning no new themes or significant insights emerged from additional interviews. The sampling approach was guided by Polkinghorne's [30] recommendation to interview 5–15 individuals who have experienced the phenomenon under study.

### 2.3. Data Collection Instrument

A semistructured interview guide was adapted from Abou Hashish and Ashour [5] and consisted of two main sections. The first section collected demographic information about the participants, including their age, nationality, educational background, job title, years of nursing experience, and years working in Saudi Arabia. The second section of the interview guide focused on open-ended, phenomenologically oriented questions designed to elicit the lived experiences of expatriate nurses concerning their migration journeys, adaptation, and retention in Saudi Arabia. These questions were framed to uncover how nurses make sense of their professional and personal transitions:

− Can you describe your experience working as a nurse in your home country and how it influenced your desire to migrate?
− What has your experience been like transitioning into the Saudi healthcare system?
− How have your experiences in Saudi Arabia influenced your decision to stay or consider leaving?
− Based on your experience, what strategies do you think would help retain expatriate nurses in Saudi hospitals?

Additional probing questions were used to encourage participants to elaborate on their experiences, such as “What do you mean?” and “Can you clarify?” Two pilot interviews were conducted to assess the clarity and appropriateness of the interview questions. These pilot interviews were used solely for refinement purposes and were not included in the final dataset or thematic analysis.

### 2.4. Ethical Considerations and Data Collection

The study adhered to the international ethical principles outlined in the Helsinki Declaration (2008). Approval was obtained from the King Abdullah International Medical Research Center (KAIMRC) Institutional Review Board under IRB (approval no. NRJ23J-271-10). Written informed consent was obtained from all participants after explaining the purpose of the study, their voluntary participation, and the right to withdraw at any time without consequences.

Participants were recruited through purposive sampling with the assistance of nurse managers at the selected hospital. Nurses who met the inclusion criteria were approached directly by the researchers, and those who agreed to participate, interviews were scheduled with them at a time convenient to them.

Data were collected using semistructured interviews, held either face-to-face or via Microsoft Teams, depending on participant's preferences and work schedules. Face-to-face interviews were conducted in the meeting room of the participants' respective units to ensure privacy and convenience. At the start of each interview, the researchers provided an overview of the study and clarified ethical considerations, including confidentiality and anonymity. The expected interview duration was 30–45 min. All interviews were audio-recorded with participant permission, and detailed field notes were documented immediately afterward to capture nonverbal cues and contextual observations.

Data collection began with two pilot interviews used to refine the interview guide; these were excluded from the final analysis. Formal data collection then proceeded until data saturation was reached, when no new themes or insights emerged, resulting in a total of 36 interviews conducted over a four-month period, from October 2024 to January 2025.

### 2.5. Data Management and Analysis

The qualitative data collected during the interviews were transcribed verbatim, ensuring that participants' voices and narratives were accurately preserved. The data were analyzed using the thematic analysis framework proposed by Braun and Clarke [31]. All authors were involved in data analysis. Initially, the researchers engaged in familiarization with the data by repeatedly reviewing the transcripts to develop an in-depth understanding of participants' narratives and contexts. This phase was followed by initial coding, during which significant statements, key concepts, and recurring phrases were identified and categorized into preliminary codes. The next step involved theme development, where recurring patterns across the data were grouped into overarching themes that encapsulated critical aspects of nurse migration and retention. These themes were then subjected to theme refinement, ensuring internal coherence and consistency while selecting illustrative participant quotations that represented the essence of each theme.

### 2.6. Trustworthiness and Rigor

Academic rigor was ensured by adhering to the four key criteria of trustworthiness: credibility, dependability, confirmability, and transferability [32]. Credibility was established through member-checking, allowing participants to validate their responses and clarify any ambiguities during data collection. Dependability was maintained by applying a consistent methodological approach across all interviews, ensuring uniformity in data collection and analysis. Confirmability was addressed through reflexivity, where the researcher kept detailed field notes documenting reflections and decisions throughout the research process, minimizing potential bias and ensuring transparency in data interpretation. Transferability was achieved by providing detailed descriptions of the research context, participant demographics, and methodological steps, allowing readers to assess the applicability of the findings to similar settings.

## 3. Results

### 3.1. Participants' Characteristics

The study included 36 expatriate nurses working in Saudi Arabia. Their ages ranged from under 30 to over 50 years, with a mean of 41.58 ± 8.00 years. On average, they had 9.15 ± 5.07 years of nursing experience and had worked in Saudi Arabia for 5.97 ± 3.95 years. The sample comprised a diverse group of nationalities, including Britain, Filipino, Malaysian, South African, Indian, and Jordanian nurses. Regarding career plans, 86.1% (*n* = 31) of nurses intended to continue working in Saudi Arabia, while 13.9% (*n* = 5) did not plan to stay. See Supporting Table 1 for detailed information about interviewed participants' characteristics.

### 3.2. Thematic Analysis Framework

**Figure 2:**
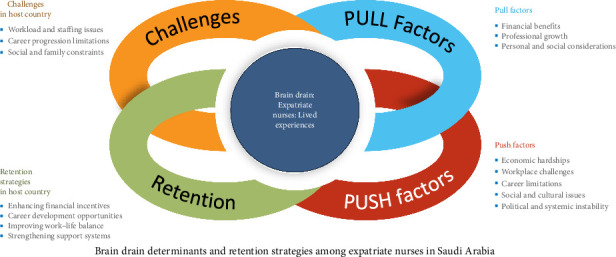
Summary of emerged themes and subthemes.

Thematic analysis of the qualitative data from expatriate nurses in Saudi Arabia revealed four major themes: push factors (reasons for migration), pull factors (attractions to Saudi Arabia), challenges faced in Saudi Arabia, and retention strategies (ways to improve retention). Within these themes, 15 subthemes were identified, reflecting various aspects of nurses' experiences. The analysis further uncovered 31 related factors, each representing specific issues or motivators that influenced nurses' migration decisions, adaptation challenges, and factors contributing to job satisfaction or dissatisfaction. While the full thematic structure includes subthemes and subfactors, this paper focuses on presenting and discussing the four main themes. See Table 1 and Figure 2 for a summary of the emerged themes, subthemes, and subfactors. Additional representative quotations for each theme are provided in Supporting Table 2.

#### 3.2.1. Theme I: Push Factors (Reasons for Migration)

Expatriate nurses identified several reasons for leaving their home countries, primarily related to economic, workplace, career, and social challenges. Economic hardships were a significant motivator, with nurses citing low wages and financial struggles as major issues. One Indian ICU nurse shared, *“Every year, inflation rises, but my salary stays the same. No matter how hard I work, my financial situation does not improve.”* Workplace challenges were also common, including staff shortages, lack of resources, and insufficient management support. As a South African surgical ward nurse explained, *“It felt like we were doing the job of three nurses at once. The patient load was unmanageable.”* A Britain charge nurse added, *“We were constantly understaffed, which meant sacrificing quality care just to get through the shift.”* An Indian staff nurse explained, *“Modern treatments and technology were only available in private hospitals, which most patients couldn't afford.”* Nurses also reported a lack of management support and recognition. A Filipino NICU nurse reinforced this by saying, *“We were expected to do more with fewer resources, and when things went wrong, nurses were the ones blamed.”*

Career limitations further pushed nurses to migrate, as opportunities for promotions and specialization were minimal. An Indian ER nurse noted, *“In my home country, promotions were based on favoritism rather than merit.”* The absence of training opportunities also played a role, with a Jordanian surgical ward nurse stating, *“We had no hospital-sponsored training, so if you wanted to learn, you had to pay out of pocket.”*

Social and systemic factors, such as negative perceptions of nursing, unstable healthcare policies, and security concerns, added to the reasons for migration. An Indian staff nurse explained, *“Nursing is not respected in my home country. People see it as a low-status job.”* A Filipino NICU nurse noted, *“My family did not support my career choice because of the social stigma surrounding nursing.”* An Indian ICU nurse explained, *“Corruption in the healthcare system meant that even basic hospital supplies were often unavailable.”*

#### 3.2.2. Theme II: Pull Factors (Attractions to Saudi Arabia)

Expatriate nurses identified several attractive features that influenced their decision to migrate to Saudi Arabia. These pull factors were categorized into financial benefits, professional growth, and personal or social considerations.

Financial benefits were the most frequently cited motivators. Participants highlighted higher salaries compared to their home countries, along with allowances that significantly reduced their living expenses. A Filipino staff nurse stated, “*The salary is competitive, and even with taxes, it is still better than what I earned at home,”* while an Indian ICU nurse shared, “*With my income here, I can send money home and still live comfortably.”* These earnings allowed many nurses to support their families abroad while maintaining a good standard of living in Saudi Arabia. In addition to salary, employer-provided housing and transportation allowances were highly valued. As one South African ER nurse explained, *“Having employer-provided housing is a huge financial relief,”* referring to the cost savings of not having to arrange or pay for accommodations independently. Another financial benefit was the end-of-service bonus, a lump-sum payment granted at the end of the employment contract, often based on years of service. A Britain nurse supervisor described this as a *“security for the future,” providing a sense of financial stability*.”

Professional growth was another compelling factor. Many nurses noted that Saudi hospitals offer access to advanced medical equipment and technology, which enhances clinical practice and learning. A Malaysian ICU nurse commented, *“The hospitals are well-equipped, and I get hands-on experience with the latest medical advancements.”* Similarly, a Jordanian surgical ward nurse stated, *“I finally feel like I can practice nursing with all the necessary tools at my disposal.”* These opportunities allowed expatriate nurses to update their skills and work in high-acuity care environments. Furthermore, opportunities for specialization and continuing education were frequently mentioned. One Filipino NICU nurse said, *“The hospital offers training programs that allow me to gain certifications,”* and a Malaysian charge nurse affirmed, *“Career progression is structured, and promotions are based on merit.”* A nurse manager from the United Kingdom described her experience, emphasizing how Western clinical standards are highly valued in Saudi specialty hospitals, *“Each employee is provided with spacious accommodation… Working at MNGHA gives me great opportunities to grow in many areas… The facilities are really good; we have a gym, swimming pools… Working abroad is excellent. … The contract includes all these benefits.”*

Personal and social considerations also played a significant role. Religious and cultural alignment was particularly meaningful for Muslim nurses, who appreciated the ability to freely practice their faith in a supportive environment. A Jordanian nurse shared, *“Being in a country where I can practice my religion freely was a major factor in my decision,”* while another nurse from Malaysia added, *“Performing Hajj and Umrah while working is an incredible opportunity.”* In addition, many expatriate nurses appreciated the sense of safety and peacefulness in Saudi Arabia. An Indian ER nurse commented, *“Living here is much safer than in my home country, where crime is high,”* and a Filipino nurse added, *“Saudi Arabia offers a peaceful environment to focus on work.”* These pull factors, financial, professional, and personal, collectively shaped the positive outlook of many expatriate nurses and strongly influenced their decision to work and remain in the Saudi healthcare system.

#### 3.2.3. Theme III: Challenges Faced in Saudi Arabia

Despite the attractive pull factors, many expatriate nurses reported notable challenges after relocating to Saudi Arabia. One of the most pressing concerns was the high patient-to-nurse ratios, which significantly increased workload and emotional strain. A Filipino ER nurse shared, *“Every shift feels like a crisis due to the shortage of nurses. We need more staff to manage patient care effectively,”* while an Indian ICU nurse added, *“The stress of handling too many patients at once affects both our well-being and the quality of care we provide.”* These comments reflect a systemic staffing issue that compromises both care quality and nurse health.

Another frequently cited challenge was inconsistent scheduling, which refers to unpredictable shift rotations, last-minute duty roster changes, and inadequate rest between shifts. Nurses reported working 12-h shifts as standard and were often expected to take on overtime or extra shifts, especially during peak admission times, staff shortages, or emergency coverage needs. A Jordanian surgical ward nurse stated, *“I rarely get a break during my shift because we are always understaffed,”* and a South African NICU nurse noted, “*We are expected to work overtime without proper compensation, which leads to burnout.”* These demanding work conditions contributed to fatigue, disrupted work–life balance, and dissatisfaction.

Regarding career progression, many expatriate nurses expressed frustration over limited leadership opportunities, often linked to institutional policies that prioritize Saudi nationals for supervisory and administrative roles. A Britain charge nurse remarked, *“No matter how many years of experience I have, I feel overlooked for promotions,”* while a Malaysian ICU nurse explained, *“Our contributions are significant, but opportunities for leadership roles are limited for expatriates.”* These limitations created a sense of stagnation, particularly for experienced expatriates with leadership potential. In addition, several nurses pointed out the lack of structured mentorship programs that could support professional development and skill advancement. For instance, a Pakistani staff nurse stated, *“I have received training certificates, but they do not seem to impact my chances for advancement,”* and a Filipino ER nurse added, *“Many skilled expatriate nurses feel stuck in their current roles due to promotion restrictions.”*

Social and family-related constraints also emerged as significant concerns. Restricted family sponsorship policies made it difficult for nurses to bring their spouses or children to live with them in Saudi Arabia, particularly under hospital-based contracts that do not offer family accommodations or visa support. An Indian surgical ward nurse expressed, *“Being away from my spouse and children for years is emotionally draining,”* and a Filipino ICU nurse emphasized, “*If family visas were easier to obtain, I would be more willing to commit long-term.”* These constraints impacted emotional well-being, caused long-term family separation, and affected retention decisions, especially for nurses who aspired to establish stable family lives in the host country.

Finally, cultural adaptation challenges were also reported, particularly during the early period of relocation. A Pakistani nurse shared, *“Adjusting to cultural norms was challenging at first, but I have adapted over time,”* reflecting the effort required to understand local customs, gender roles, and religious practices. Some participants suggested more formal orientation and community support initiatives to ease the transition. As one Jordanian nurse manager noted, “*There should be more community support programs for expatriate nurses,”* highlighting the need for systems that promote smoother integration into the work and social environment. These cultural challenges, although eventually manageable for many, initially contributed to emotional stress and feelings of isolation.

#### 3.2.4. Theme IV: Retention Strategies

Participants proposed several strategies to improve nurse retention in Saudi Arabia, addressing financial, professional, personal, and systemic needs. A major recommendation was to enhance financial incentives, including performance-based bonuses and salary adjustments. An Indian ER nurse shared, *“Retention bonuses for nurses who have been here for over 5 years would encourage long-term commitment,”* while a South African charge nurse suggested, *“Higher salaries should reflect the cost of living and responsibilities of our job.”* Some also emphasized the importance of adjusting housing and transportation allowances to ease the financial burden of living alone abroad. A Britain ICU nurse noted, *“If housing and transportation allowances were increased, it would ease financial burdens.”*

Interestingly, one Filipino nurse pointed out, *“Offering child education benefits would make long-term employment more attractive.”* While most expatriate nurses are unable to sponsor their families under typical contracts, this comment reflects the reality that some nurses are able to bring dependents under specific job categories or hope to do so if policies change. This perspective highlights the desire for more family-inclusive policies and suggests that expanding family sponsorship and support services could positively influence retention.

In addition to financial concerns, participants strongly advocated for career development opportunities, including structured mentorship, specialization tracks, and access to advanced education. A Jordanian nurse manager stated, *“Providing structured career paths for expatriates would make staying in Saudi Arabia more appealing,”* while a Malaysian staff nurse added, *“Mandatory professional development programs should be accessible to all nurses.”* Other suggestions included leadership development and scholarship opportunities. For example, a Pakistani surgical ward nurse noted, *“Leadership training should be introduced to help nurses transition into higher roles,”* and an Indian NICU nurse suggested, *“Scholarships for higher education would motivate more nurses to stay.”*

Improving work –life balance was another priority. Many nurses described the need for flexible scheduling, which refers to implementing predictable shift rotations, avoiding excessive consecutive night shifts, and allowing more control over shift preferences when possible. A Filipino ER nurse explained, *“A better shift rotation policy would help nurses maintain a work –life balance,”* while another Filipino charge nurse shared, “*More leave options would help prevent burnout and improve mental well-being.”* These insights reflect the demand for healthier scheduling practices that account for physical and emotional recovery, especially for nurses working 12-h shifts.

Participants also emphasized the importance of mental health support and family-friendly workplace policies. A Britain nurse educator suggested, *“Providing mental health support for nurses would improve job satisfaction,”* and an Indian ICU nurse added, *“Expanding family sponsorship options would increase expatriate nurse retention.”*

Finally, nurses called for stronger support systems, including better integration into the hospital environment and greater inclusion in organizational decision-making. A Pakistani senior nurse stated, *“Expatriate nurses should have representation in hospital leadership committees,”* and a Jordanian nurse manager emphasized, *“More cultural integration programs would help new nurses settle in faster.”* Suggestions also included forming dedicated human resource (HR) teams for expatriates and expanding mentorship support for new hires, aiming to foster a more inclusive, stable, and supportive professional environment.

In summary, Figure 2 presents a visual overview of these findings, mapping how each theme and its related subthemes interconnect to form a comprehensive picture of expatriate nurses' experiences in Saudi Arabia. It illustrates the dynamic interaction between premigration push factors, postmigration pull factors, the challenges encountered within the Saudi healthcare system, and the strategies suggested by participants to improve retention. This framework serves as a conceptual foundation for understanding the multifaceted nature of nurse migration and retention from the perspective of those directly affected.

## 4. Discussion

The findings of this study align with existing literature on the brain drain phenomenon among nurses, confirming that migration decisions are shaped by a combination of push and pull factors. In addition to identifying these drivers of migration, this study uniquely explored the challenges expatriate nurses face in Saudi Arabia, the strategies that may enhance their retention and career intentions.

### 4.1. Push Factors and Nurses' Migration

The results demonstrate that economic challenges are a predominant driver of expatriate nurse migration. Many participants cited inadequate salaries in their home countries, pushing them to seek better financial opportunities abroad. This aligns with findings by Abou Hashish et al. [3] and Kadel and Bhandari [4], which identified low income and financial insecurity as the most cited push factors contributing to nurse brain drain in LMICs. Poor working conditions were another critical factor. Many expatriate nurses in this study reported chronic understaffing, long hours, and lack of resources in their home systems, experiences that echo those described by Pretorius [14] in South Africa and by Konlan et al. [2], who found that nurses in LMICs were driven to migrate due to outdated equipment, poor infrastructure, and weak healthcare governance. These structural issues created a sense of professional stagnation and emotional fatigue, prompting nurses to seek environments with better support and resources.

Prioritizing drivers of nursing migration, Afshari et al. [33] emphasized that economic factors were the primary catalysts, aligning with international evidence suggesting that economic disparities, such as inadequate compensation, high living costs, and the lure of better opportunities abroad, are among the most influential push factors for nurses globally. These were further broken down into subcategories including wage dissatisfaction, financial insecurity, and the burden of supporting families, which were also echoed by participants in this study.

In contrast, the few participants from high-income countries, such as the United Kingdom, described a different set of migration drivers. While financial benefits were still appreciated, their push factors centered more around systemic burnout, workplace dissatisfaction, and a desire for new international experiences rather than economic hardship. For example, United kingdom-based nurses described bureaucratic overload, staff attrition, and increased emotional exhaustion as demotivating factors, an interpretation supported by literature indicating that nurses from HICs often cite poor staffing ratios, professional burnout, and dissatisfaction with management structures as key reasons for migration [17, 34].

Moreover, limited career advancement was consistently reported across both LMIC and HIC participants, although the context differed. Nurses from LMICs highlighted a lack of merit-based promotion and out-of-pocket training costs, while HIC nurses referred to glass ceilings and bureaucratic limitations. This finding is in line with Alameddine et al. [35] and Okafor and Chimereze [18], who noted that career stagnation plays a critical role in both intra- and interregional migration. Social and systemic influences, including negative perceptions of nursing, governance instability, and safety concerns, also contributed to migration decisions. Blau et al. [36] emphasized how professional misrepresentation and low public regard can devalue the nursing role. Similarly, Abou Hashish and Ashour [5] linked societal undervaluation of nurses to increased migration intent.

In the Saudi context, studies confirm that similar dissatisfaction exists among expatriate nurses. For instance, Alreshidi et al. [23] and Almansour et al. [8] found that workload stress, perceived inequity, and lack of empowerment contributed to turnover among non-Saudi nurses. These findings illustrate how global push factors intersect with local dynamics and suggest that retention strategies in Saudi Arabia must consider both the diverse backgrounds and differentiated motivations of the expatriate workforce.

### 4.2. Pull Factors and Attraction to Saudi Arabia

The findings of this study revealed several interrelated factors that attracted expatriate nurses to Saudi Arabia. These included financial incentives, opportunities for professional development, religious and cultural alignment, and workplace stability. While these pull factors have been consistently reported in the literature, their manifestation in the Saudi context reflects a unique combination of policy, cultural factors, and institutional practice.

Economic opportunity emerged as one of the most immediate and compelling motivators, particularly for nurses from LMICs. Participants noted that salary structures in Saudi Arabia, along with additional benefits such as housing, transportation, and performance-based bonuses, provided a degree of financial security and upward mobility that was not available in their home countries. This finding is consistent with the work of Almansour et al. [8], who identified comprehensive compensation packages as a major draw for healthcare professionals migrating to Saudi Arabia. Afshari et al. [33] similarly emphasized that inadequate wages and high living costs in origin countries are among the most common global drivers of nurse migration.

Professional development and structured advancement opportunities were also highlighted as significant pull factors. Participant nurses reported access to advanced healthcare technologies, continuing education programs, and defined career paths as central to their decision to relocate. These findings reflect the goals outlined in Saudi Vision 2030, which prioritizes workforce development and the localization of healthcare expertise [37]. Alreshidi et al. [23], Almansour et al. [12], and Mazibu et al. [38] also emphasized that offering professional growth opportunities is instrumental in attracting and sustaining a qualified and expatriate nursing workforce.

Religious and cultural alignment was another prominent attraction, especially among Muslim nurses. Many participants expressed a deep appreciation for being able to practice their faith freely and to perform religious rites such as Hajj and Umrah while living and working in Saudi Arabia. This spiritual compatibility contributed not only to their initial interest in relocating but also to a sense of cultural belonging once employed. These findings are supported by earlier research by Abou Hashish and Ashour [5], who found that religious proximity and cultural familiarity were integral to destination choice among Muslim expatriate nurses.

In addition to financial, professional, and spiritual factors, many participants appreciated the social and political stability of Saudi Arabia. Several nurses who had previously worked in or migrated from conflict-prone or economically unstable regions emphasized the appeal of Saudi Arabia's secure, structured, and well-governed healthcare institutions. These sentiments mirror prior studies that identified safety, organizational reliability, and predictable work environments as important migration factors for nurses from unstable home settings [2, 14].

Although many of these pull factors were widely shared, their relative importance varied across nationalities and regions. Nurses from countries such as India, the Philippines, Pakistan, and Sudan predominantly cited financial stability, family support, and long-term security as central drivers. Meanwhile, nurses from high-income countries, such as the United Kingdom, placed more emphasis on professional repositioning, access to leadership roles, and the opportunity to work in an internationally respected system (Almansour et al. [8]). Some participants noted that Western-trained nurses were more likely to be placed in senior or executive nursing roles, particularly in high-status institutions such as the Ministry of National Guard Health Affairs, offering not only enhanced authority but also higher salaries and benefits. These pathways into leadership were described as both attractive and professionally validating.

Nurses from middle-income countries, such as Jordan, described a dual motivation, combining economic aspirations with cultural and religious affinity. This layered motivation structure reflects how migration drivers are shaped not only by financial need or systemic gaps at home but also by the perceived compatibility between personal values and the host country's institutional culture [2].

While some of these factors, such as salary and advancement opportunities, also appear in the context of nurse retention, their role in the migration decision is distinct. In this analysis, they are understood as preemployment motivators that shape the decision to leave one's country and choose Saudi Arabia as a destination. This distinction helps delineate between what attracts nurses initially and what sustains them long-term, which is further explored in subsequent sections [39].

### 4.3. Challenges Faced by Expatriate Nurses

Despite the attractive pull factors, many expatriate nurses reported encountering significant challenges after relocating to Saudi Arabia. Common issues included social and cultural constraints, family separation, workplace dissatisfaction, and language and integration difficulties, all of which contributed to some participants considering early departure.

Recent Saudi studies offer strong support for these findings. Almansour et al. [8, 12] documented that limited Arabic proficiency, rigid work cultures, and separation from family negatively impacted expatriate nurses' adaptation, particularly affecting younger staff, non-Muslims, and those without Arabic fluency. A cross-sectional survey found that nearly 40% of non-Saudi healthcare workers reported cultural adaptation as a major stressor, significantly correlated with turnover intention [11]. Earlier international work, such as Pung and Goh [40] and Mokoena [41], similarly identified communication barriers, legal complexities, and cultural misalignments as persistent challenges in foreign nursing environments.

On the organizational front, workload pressures, limited career progression, and role stagnation emerged as critical themes. Almansour et al. [42] revealed that expatriate nurses in Saudi settings often expressed dissatisfaction with workload and lack of career mobility despite positively rating collegial relationships. Almansour et al. [12] further noted that restrictions on higher education and job security, compounded by complex residency and visa systems, frequently forced skilled nurses to reconsider their tenure. These findings align with broader research [2, 22] showing that migrant healthcare providers often face professional deskilling, discrimination, and unmet expectations upon migrating.

Importantly, negative experiences were not uniform across nationalities. Nurses from LMICs (e.g., the Philippines, India, and Pakistan) were especially affected by language limitations, regulatory burdens, and family separation. Konlan et al. [2] and Ejebu et al. [39] concur with these findings. These impacts were magnified among non-Muslim and non-Arabic-speaking nurses, many of whom struggled more during orientation and integration in clinical and social environments. By contrast, nurses from high-income countries, such as the United Kingdom, often had stronger English proficiency and greater cultural familiarity, enabling them to assimilate more quickly. However, they reported frustrations related to workload management, professional status uncertainty, and organizational rigidity in systems heavily oriented toward nationalization. Jordanian nurses, meanwhile, reported more complex cultural–cultural dynamics, where cultural similarity facilitated adaptation, but economic mismatch still drove dissatisfaction.

Saudi-based research further highlights the institutional impact of these challenges. For example, a mixed-methods study by Alreshidi et al. [23] found that nearly 55% of foreign nurses in Saudi public hospitals considered leaving within 2 years due to unsustainable work conditions, poor work–life balance, and inadequate cultural orientation programs. These findings underscore how facing unmet expectations after migration, such as a lack of tailored integration support or professional recognition, can undermine the very pull factors that attracted nurses in the first place. It is critical, therefore, to analyze these challenges as dynamic hurdles that disproportionately affect certain nationalities, career stages, and demographic groups. While the initial decision to migrate may be driven by economic or career opportunity, the decision to stay or return hinges on the ability of the host system to support meaningful adaptation, career progression, and culturally respectful environments. If expatriate nurses arrive with expectations influenced by global standards and personal ambitions, failure to meet these expectations, especially in visa facilitation, scheduling flexibility, and career inclusion, can quickly erode retention potential [43, 44]. This complex interplay highlights the importance of proactive strategies, such as language support, mentorship programs, enhanced cultural induction, diversified leadership pathways, and streamlined residency policies, to reinforce expatriate nurse integration and tenure in Saudi Arabia.

### 4.4. Retention Strategies: Addressing Expatriate Nurse Challenges in Saudi Arabia

In response to the diverse challenges they experienced, including professional stagnation, workload burden, cultural adaptation difficulties, and family separation, participants proposed a range of strategies to enhance expatriate nurse retention. These recommendations highlight both systemic and individualized solutions that reflect the complexity of the Saudi healthcare environment and the heterogeneity of its nursing workforce.

Financial incentives were among the most consistently emphasized strategies. Nurses from LMICs viewed enhanced salary packages, performance-based bonuses, and increased housing allowances as essential to their long-term commitment. These economic concerns directly echoed the initial push factors that led many of them to migrate in the first place. Their responses align with national data indicating that over 80% of expatriate nurses in Saudi Arabia identify financial concerns as their primary source of dissatisfaction [23]. In institutions where cost-of-living adjustments were infrequent or unclear, participants felt undervalued, and several noted they were considering contract termination. Alanazi et al. [45] revealed that around 84% of nurses cited fair compensation as a key retention factor, with over half specifically mentioning housing benefits. In this regard, offering competitive, transparent, and regularly reviewed compensation packages would not only mitigate financial stress but also reinforce a sense of professional worth, particularly for nurses supporting extended families abroad.

In addition to financial recognition, many participants expressed a strong desire for meaningful professional development. Challenges related to limited leadership roles and lack of promotion opportunities were frequently cited, especially among experienced nurses who felt that their contributions were overlooked due to nationality or systemic bias. Western-trained nurses, including those from the United Kingdom, reported being more likely to access senior or managerial roles, which they found highly motivating. This contrasted with some participants from LMICs who described being “stuck” in bedside roles regardless of tenure or performance. These accounts reflect existing research by Almansour et al. [12] and Viken et al. [46], who found that expatriate nurses in Saudi Arabia often encounter unclear promotion pathways and limited participation in decision-making processes.

In this context, structured career ladders, equitable access to continuing education, and mentorship programs were recommended as key solutions. Participants suggested leadership training workshops, tailored specialization tracks, and scholarship access for long-term nurses to support advancement. Institutional support for these strategies would not only boost morale but also foster a culture of inclusivity and growth [47]. Importantly, such development programs must be adapted to meet the distinct goals of different groups: early-career LMIC nurses may prioritize certification and skills-building, while senior nurses from high-income countries may be more drawn to executive-level responsibilities and strategic engagement.

Work–life balance also emerged as a critical area for retention intervention. Many participants, particularly those working in intensive care or emergency settings, reported long shifts, unexpected overtime, and inadequate rest periods. As one nurse put it, “Every shift feels like a crisis.” The emotional and physical toll of these workloads, combined with separation from family, created cumulative stress. Nurses with young children or dependents abroad noted that the absence of flexible scheduling contributed to burnout and made it harder to remain committed to their roles.

To address these issues, participants recommended improvements such as more predictable rotation schedules, mandatory rest periods, and capped overtime. These strategies are consistent with Alsayed et al. [48], who emphasized the role of staffing and scheduling policies in promoting nurse well-being and retention. Given that many expatriate nurses are separated from their families for extended periods, work–life balance initiatives are not merely about personal comfort, they are central to emotional resilience and job sustainability.

Another major theme was the need for better social and cultural integration. As identified earlier, challenges such as language barriers, unfamiliar norms, and exclusion from decision-making roles left many expatriate nurses feeling isolated. Participants recommended structured orientation programs focusing on Arabic language basics, cultural expectations, and professional conduct in the Saudi context. Furthermore, several nurses emphasized the value of being represented in hospital governance structures, suggesting that expatriate advisory committees could help voice concerns, improve transparency, and build trust.

Recent national efforts to localize the workforce have sometimes inadvertently created tensions between Saudi and expatriate staff, making it all the more important to foster inclusive environments that support mutual respect. Cultural sensitivity training for all staff and formal inclusion of expatriate nurses in planning and policy discussions could bridge this gap. In discussing Saudi nurses' retention, Fabiana et al. [49] emphasized that adapting healthcare policies in Saudi Arabia to better align with cultural and religious expectations is essential for improving nurse retention. They argue that policies bridging the gap between female nurses' family obligations and professional roles can foster workplace equality while honoring societal norms. Such culturally sensitive approaches are particularly important in supporting the largely female nursing workforce and ensuring their sustained engagement and job satisfaction. Almansour et al. [8] reported that such participatory strategies are linked to higher job embeddedness and retention among foreign health workers.

Finally, participants urged the government and health institutions to revise policies on family sponsorship. Several nurses shared emotional accounts of being separated from spouses and children for years. This strain was particularly acute for mid-career nurses from LMICs who otherwise expressed satisfaction with their jobs but felt emotionally exhausted by isolation. The authors in reference [49] identified facilitating family visas, offering schooling benefits for children, and supporting long-term residency as crucial retention factors. Taken together, these strategies point to the need for context-sensitive, multilevel solutions. As Ejebu et al. [39] noted, a “one-size-fits-all” approach is insufficient to support a nursing workforce characterized by such diverse motivations, experiences, and needs. Retention efforts must be responsive to the unique concerns of different demographic groups, whether that means financial stability for nurses from the Philippines or India, career advancement for Britain or South African professionals, or cultural and religious alignment for Jordanian or Malaysian staff.

In conclusion, improving expatriate nurse retention in Saudi Arabia requires more than addressing surface-level dissatisfaction. It demands systemic investment in fair compensation, transparent career mobility, flexible work structures, cultural integration, and emotional support. These tailored interventions not only respond directly to the challenges identified in this study but also contribute to a more sustainable, resilient healthcare workforce aligned with the goals of Saudi Vision 2030. In this context, Albalawi et al. [50] stressed the need for stakeholders to improve the work environment by enhancing nurses' job satisfaction and overall quality of life. A critical component involves reassessing salary structures to ensure competitive remuneration. In addition, improving housing conditions and expanding benefits can significantly influence retention. For expatriate nurses, targeted support in resolving immigration-related challenges is essential to reduce psychological stress and personal instability. Addressing these interconnected factors not only strengthens workforce stability but also curbs outward migration and minimizes the high costs associated with continuous international recruitment.

## 5. Strengths and Limitations of the Study

### 5.1. What This Study Adds Compared to Previous Research

This study provides a comprehensive exploration of the brain drain phenomenon among expatriate nurses in Saudi Arabia. Unlike previous research that predominantly focused on migration drivers alone, it also examines postmigration realities, specifically the challenges nurses face and the factors that influence their decisions to stay or leave, thus offering critical insights for both recruitment and retention strategies. A key strength lies in its in-depth thematic analysis, which categorizes nurses' lived experiences into four interrelated themes: push factors, pull factors, challenges, and retention strategies. This framework enables a holistic understanding of not only migration intentions but also the structural and emotional factors that shape long-term career decisions.

Another distinguishing feature of this study is the inclusion of a demographically and nationally diverse sample, encompassing nurses from Britain, the Philippines, India, South Africa, Malaysia, and Jordan. This diversity offers a broader comparative perspective on how nationality, training background, and personal values influence migration and retention. Unlike studies that generalize expatriate experiences, this study acknowledges intragroup differences and provides tailored recommendations for workforce planning. By integrating both premigration and postmigration variables, the study bridges a critical gap between global migration research and practical policy design in the context of the Saudi healthcare system. The findings are therefore applicable not only to Saudi Arabia but also to similar healthcare systems relying on a multinational nursing workforce.

### 5.2. Limitations

Despite these strengths, several limitations should be acknowledged. First, the study only included expatriate nurses working in Saudi Arabia, which may make it hard to apply the results to other places, such as nurses in other Gulf Cooperation Council (GCC) countries, non-GCC Middle Eastern countries, or Western healthcare systems that have different migration patterns. Second, data collection occurred at a single point, and the absence of longitudinal follow-up limits the ability to assess how nurses' migration motives, job satisfaction, and retention outlook may evolve.

Third, as a qualitative study, the findings are based on subjective self-reporting and interpretive analysis, which may introduce bias despite methodological rigor. While efforts were made to include variation in nationality, role, and clinical setting, participants were recruited from a single tertiary hospital in the western region of Saudi Arabia. As healthcare practices and expatriate integration policies can vary by region or institution, the experiences described may not be fully representative of all expatriate nurses working across the Kingdom.

Moreover, the inclusion of participants from countries with markedly different healthcare systems, such as the United Kingdom (a high-income, publicly funded model) and Jordan (a middle-income, mixed model), not only adds valuable diversity but also complicates uniform interpretation. Participants' expectations, adaptability, and long-term career decisions may be shaped as much by home-country factors as by host-country conditions. This heterogeneity enriches the data but presents limitations in drawing universally applicable conclusions. Future studies are needed to explore nationality-specific trends, particularly using stratified or comparative designs, and to evaluate tailored retention strategies that reflect the complex intersection of cultural, economic, and professional factors influencing expatriate nurse mobility.

## 6. Conclusion

This study provides an in-depth exploration of the brain drain phenomenon among expatriate nurses in Saudi Arabia by examining the interplay between push factors, pull factors, postmigration challenges, and retention strategies. The findings reveal that economic hardship, limited career advancement, and difficult working conditions in home countries are key drivers of migration. Conversely, financial incentives, professional opportunities, and cultural or religious alignment serve as strong motivators for nurses to work in Saudi Arabia.

However, the study also highlights persistent challenges faced after migration, including heavy workloads, restricted leadership opportunities, and social integration barriers. These challenges vary across nationalities, reflecting the diverse experiences and expectations of expatriate nurses. By capturing these multifaceted experiences, the study contributes to a deeper understanding of why nurses choose to migrate, remain, or leave and emphasizes the importance of developing tailored, equitable workforce strategies to support retention. These insights are especially relevant for healthcare systems that rely heavily on foreign-trained nurses and seek to ensure workforce sustainability in the face of global nursing shortages.

## 7. Implications of the Study

### 7.1. Implications for Practice and Nursing Management

− This study underscores the critical role of nursing leadership, hospital administration, and national health policymakers in shaping sustainable strategies for retaining expatriate nurses. Given the centrality of workload, career progression, and integration challenges identified in this study, healthcare institutions must adopt multilevel, culturally responsive interventions.
− Effective staffing models should ensure safe patient-to-nurse ratios and predictable scheduling to minimize burnout and preserve work–life balance. This is particularly important in high-demand units such as critical care and emergency departments where most expatriate nurses are deployed. Nurse managers should regularly assess staffing adequacy and foster supportive team dynamics to reduce emotional exhaustion.
− Career development structures must be made transparent and inclusive, allowing expatriate nurses equitable access to mentorship, training, and promotion. Institutions should establish clear pathways for leadership advancement, especially for experienced nurses from high-income countries who may be positioned for managerial or advisory roles. Aligning professional development opportunities with nurses' prior experiences and career goals will strengthen engagement and reduce attrition.
− From a policy standpoint, national healthcare authorities should prioritize family reunification and visa flexibility as integral components of retention. Enhancing access to family sponsorship and providing child education benefits may reduce emotional strain for mid-career expatriates from LMICs. In parallel, streamlined and culturally oriented induction programs, including basic Arabic language support and workplace orientation, are vital to accelerate adjustment and boost early-stage retention.
− Equitable compensation remains essential. Regular salary benchmarking, housing allowance reviews, and the introduction of performance-based retention packages aligned with international norms can enhance perceived fairness and motivation. These financial measures must be complemented by nonmonetary incentives such as inclusion in hospital governance and peer recognition programs.
− Finally, promoting a culture of inclusivity through cultural sensitivity training for both local and expatriate staff can reduce workplace tensions and improve interprofessional collaboration. Ensuring ongoing access to clinical training, especially in digital health and advanced technologies, will support continuous professional growth, reinforcing expatriate nurses' sense of value and career fulfillment within Saudi Arabia's evolving healthcare system.

### 7.2. Recommendations for Future Research

To deepen understanding of expatriate nurse retention, future research should adopt longitudinal designs to track career trajectories and explore how motivations and challenges evolve over time. Long-term studies could clarify whether initial retention strategies remain effective as personal and professional circumstances change.

Comparative research across different regions within Saudi Arabia, and across GCC countries, would provide insight into how regional variations in policy, workplace culture, and healthcare system maturity influence expatriate nurse experiences. Such cross-national studies could inform harmonized but flexible retention policies within the region.

In addition, future studies should disaggregate findings by nationality, career stage, and clinical specialty to allow for more targeted policy design. Exploring how nurses from diverse cultural and economic backgrounds interpret fairness, job satisfaction, and belonging will be critical to developing nuanced retention interventions. Mixed-methods approaches that combine quantitative turnover data with in-depth qualitative perspectives can offer a holistic understanding of the expatriate nursing workforce across settings.

## Figures and Tables

**Table 1:** Summary of emerged themes, subthemes, and subfactors.

Theme (4)	Subtheme (15)	Subfactors (31)
I. Push factors	1. Economic hardships 2. Workplace challenges 3. Career limitations 4. Social and cultural issues 5. Political and systemic instability	− Low wages and financial struggles − Limited financial incentives − Staff shortages and excessive workload − Lack of resources and outdated equipment − Lack of management support and recognition − Limited promotions and career stagnation − Lack of training opportunities − Negative public image of nursing − Work–life balance challenges − Unstable healthcare policies − Security concerns in home countries

II. Pull factors	6. Financial benefits 7. Professional growth 8. Personal and social considerations	− Higher earnings and benefits − Housing and transport allowances − Access to advanced medical equipment − Opportunities for specialization − Religious and cultural alignment − Social integration and safety

III. Challenges faced in Saudi Arabia	9. Workload and staffing issues 10. Recognition and career progression 11. Social and family constraints	− High patient-to-nurse ratios −Inconsistent scheduling − Limited leadership roles for expatriates − Need for structured mentorship programs − Restricted family sponsorship − Adjusting to cultural norms and restrictions

IV. Retention strategies	12. Enhancing financial incentives 13. Career development opportunities 14. Improving work–life balance 15. Strengthening support systems	− Performance-based bonuses − Regular salary adjustments − Providing structured mentorship − Expanding training and specialization options − Flexible scheduling − More family-friendly policies − Better integration of expatriate nurses − More inclusive decision-making processes

## Data Availability

Data are available on request from the authors.

## Supporting Information

The following supporting files are provided with this manuscript:

Supporting Table 1: Characteristics of interviewed expatriate nurses, including demographic and professional background details.

Supporting Table 2: Main themes, subthemes, related factors, and quotations of brain drain among expatriate nurses in Saudi Arabia.

COREQ checklist: The Consolidated Criteria for Reporting Qualitative Research (COREQ) guidelines checklist is followed in reporting the study findings. This is performed to ensure methodological rigor.
