# Supplementary material for: Brain Drain and Retention Strategies: Lived Experience of Expatriate Nurses in Saudi Arabia: Challenges and Implications
Source: J Nurs Manag. 2025 Aug 6;2025:9947313. doi: 10.1155/jonm/9947313 (PMC12349981; doi:10.1155/jonm/9947313)
Supplement: Supporting Information — Additional supporting information can be found online in the Supporting Information section. [file 9947313.f1.zip › Supplemntary tables Brain drain 2025.docx]

# Supplementary Table 1: Demographic and Professional Characteristics of participated Expatriate Nurses (N=36)

| **Demographic Data** | **No** | **%** |
| --- | --- | --- |
| **Age** |  |  |
| <30 | 4 | 11.1 |
| 30-39 | 8 | 22.2 |
| 40-49 | 16 | 44.4 |
| ≥50 | 8 | 22.2 |
| Mean ± SD | 41.58 ± 8.00 | |
| **Nationality** |  |  |
| British | 6 | 16.7 |
| Pakistani | 6 | 16.7 |
| Malaysian | 6 | 16.7 |
| Filipino | 6 | 16.7 |
| Indian | 4 | 11.1 |
| Jordanian | 3 | 8.3 |
| Indonesian | 3 | 8.3 |
| South African | 3 | 8.3 |
| **Current Position** |  |  |
| Nurse Supervisors | 2 | 2.8 |
| Nurse Manager | 7 | 19.4 |
| Senior Nurse | 6 | 16.7 |
| Charge Nurse | 6 | 16.7 |
| Staff Nurse | 16 | 13.9 |
| **Years of Experience in Nursing** |  |  |
| 7-10 years | 11 | 30.6 |
| 11-15 years | 8 | 22.2 |
| 1-3 years | 6 | 16.7 |
| 4-6 years | 6 | 16.7 |
| 16+ years | 5 | 13.9 |
| Mean ± SD | 9.15 ± 5.07 | |
| **Years of Working in Saudi Arabia** |  |  |
| 4-6 years | 9 | 25.0 |
| 7-10 years | 8 | 22.2 |
| 1-3 years | 7 | 19.4 |
| 11+ years | 7 | 19.4 |
| 6 months<1 year | 5 | 13.9 |
| Mean ± SD | 5.97 ± 3.95 | |
| **Do you plan to continue your career in Saudi Arabia** |  |  |
| Yes | 31 | 86.1 |
| No | 5 | 13.9 |
| **Main reason influencing decision to stay or leave Saudi Arabia (31)** |  |  |
| Financial incentives | 31 | 30.8 |
| Career growth opportunities | 16 | 44.4 |
| Work environment and job satisfaction | 11 | 30.6 |
| Family and social reasons | 11 | 30.6 |
| Cultural adaptation and lifestyle preferences | 8 | 22.2 |
| **Reasons for not continuing career in Saudi Arabia (5)** |  |  |
| Limited career growth opportunities | 5 | 13.9 |
| Family relocation or preferences | 3 | 8.3 |
| Workplace dissatisfaction | 3 | 8.3 |
| Cultural adaptation challenges | 2 | 2.8 |

# Supplementary Table 2: Final Themes, Subthemes, Related Factors, and Quotations of brain drain among expatriate nurses in Saudi Arabia

| **Theme** | **Subtheme** | **Related Factors** | ***Representative Quotations*** |
| --- | --- | --- | --- |
| 1. **I- Push Factors (Reasons for Migration)** | 1. Economic Hardships | 1. Low wages and financial struggles | *“After paying rent and utilities, my salary back home was barely enough for food and transportation.”- Filipino Nurse, Staff Nurse  “Every year, inflation rises, but my salary stays the same. No matter how hard I work, my financial situation does not improve.” - Indian Nurse, ICU Nurse* |
|  |  | 1. Limited financial incentives | *“Overtime pay was minimal, and we were often forced to work extra hours without proper compensation.”- South African Nurse, ER Nurse  “Bonuses and health benefits in my country were either inconsistent or nonexistent. I wanted a job where I felt financially secure.”- Jordanian Nurse, Senior Nurse* |
|  | 1. Workplace Challenges | 1. Staff shortages and excessive workload | *“It felt like we were doing the job of three nurses at once. The patient load was unmanageable.”- South African Nurse, Surgical Ward  “We were constantly understaffed, which meant sacrificing quality care just to get through the shift.”- British Nurse, Charge Nurse* |
|  |  | 1. Lack of resources and outdated equipment | *“There were times when we had to reuse disposable items because the hospital ran out of supplies.”- Malaysian Nurse, Critical Care Nurse  “Modern treatments and technology were only available in private hospitals, which most patients couldn’t afford.”- Indian Nurse, Staff Nurse* |
|  |  | 1. Lack of management support and recognition | *“We had no voice in decision-making. Nurses’ opinions were ignored even when we raised valid safety concerns.”- Filipino Nurse, ICU Nurse  “We were expected to do more with fewer resources, and when things went wrong, nurses were the ones blamed.”- Filipino Nurse, NICU Nurse* |
|  | 1. Career Limitations | 1. Limited promotions and career stagnation | *“In my home country, promotions were based on favoritism rather than merit.”- Indian Nurse, ER Nurse  “There was no clear path for career growth. I had been in the same position for years with no opportunity for specialization.”- South African Nurse, Nurse Supervisor* |
|  |  | 1. Lack of training opportunities | *“We had no hospital-sponsored training, so if you wanted to learn, you had to pay out of pocket.”- Jordanian Nurse, Surgical Ward  “New graduates were given no mentorship, and experienced nurses had no access to advanced certifications.”- British Nurse, Nurse Educator* |
|  | 1. Social and Cultural Issues | 1. Negative public image of nursing | *“Nursing is not respected in my home country. People see it as a low-status job.”- Indian Nurse, Staff Nurse  “My family did not support my career choice because of the social stigma surrounding nursing.”- Filipino Nurse, NICU Nurse* |
|  |  | 1. Work-life balance challenges | *“Long shifts and mandatory overtime meant I had no time for my family.”- Bangladeshi Nurse, Staff Nurse  “There was no support for working mothers, making it difficult to balance work and family life.”- Malaysian Nurse, Charge Nurse* |
|  | 1. Political and Systemic Instability | 1. Unstable healthcare policies | *“Frequent policy changes and mismanagement made it hard to plan for the future.”- Jordanian Nurse, Nurse Manager  “Corruption in the healthcare system meant that even basic hospital supplies were often unavailable.”- Indian Nurse, ICU Nurse* |
|  |  | 1. Security concerns in home countries | *“I never felt safe at work. Violence against healthcare workers is increasing.”- Indian Nurse, ER Nurse  “Healthcare workers were often harassed, and there was no protection for us.”- South African Nurse, Surgical Ward* |
| 1. **II- Pull Factors (Attractions to Saudi Arabia)** | 1. Financial Benefits | 1. Higher earnings and benefits | *“The salary is competitive, and even with taxes, it is still better than what I earned at home.”- Filipino Nurse, Staff Nurse  “With my income here, I can send money home and still live comfortably.”- Indian Nurse, ICU Nurse* |
|  |  | 1. Housing and transport allowances | *“Having employer-provided housing is a huge financial relief.”- South African Nurse, ER Nurse  “The end-of-service bonuses provide security for the future.”- British Nurse, Nurse Supervisor* |
|  | 1. Professional Growth | 1. Access to advanced medical equipment | *“The hospitals are well-equipped, and I get hands-on experience with the latest medical advancements.”- Malaysian Nurse, ICU Nurse  “I finally feel like I can practice nursing with all the necessary tools at my disposal.”- Jordanian Nurse, Surgical Ward* |
|  |  | 1. Opportunities for specialization | *“The hospital offers training programs that allow me to gain certifications.”- Filipino Nurse, NICU Nurse  “Career progression is structured, and promotions are based on merit.”- Malaysian Nurse, Charge Nurse*  *,“Each employee is provided with spacious accommodation… Working at MNGHA gives me great opportunities to grow in many areas… the facilities are really good; we have a gym, swimming pools… And all this is provided in the contract.” British Nurse manager* |
|  | 1. Personal and Social Considerations | 1. Religious and cultural alignment | *“Being in a country where I can practice my religion freely was a major factor in my decision.”- Jordanian Nurse, Surgical Ward  “Performing Hajj and Umrah while working is an incredible opportunity.”- Malaysian Nurse, Staff Nurse* |
|  |  | 1. Social integration and safety | *“Living here is much safer than in my home country, where crime is high.”- Indian Nurse, ER Nurse  “Saudi Arabia offers a peaceful environment to focus on work.”*  *- Filipino Nurse, Staff Nurse* |
| 1. **III- Challenges Faced in Saudi Arabia** | 1. Workload and Staffing Issues | 1. High patient-to-nurse ratios | *“Every shift feels like a crisis due to the shortage of nurses. We need more staff to manage patient care effectively.”*  *- Filipino Nurse, ER Nurse  “The stress of handling too many patients at once affects both our well-being and the quality of care we provide.”- Indian Nurse, ICU Nurse* |
|  |  | 1. Inconsistent scheduling | *“I rarely get a break during my shift because we are always understaffed.”- Jordanian Nurse, Surgical Ward  “We are expected to work overtime without proper compensation, which leads to burnout.”- South African Nurse, NICU Nurse* |
|  | 1. Recognition and Career Progression | 1. Limited leadership roles for expatriates | *“No matter how many years of experience I have, I feel overlooked for promotions.”- British Nurse, Charge Nurse  “Our contributions are significant, but opportunities for leadership roles are limited for expatriates.”- Malaysian Nurse, ICU Nurse* |
|  |  | 1. Need for structured mentorship programs | *“I have received training certificates, but they do not seem to impact my chances for advancement.”- Pakistani Nurse, Staff Nurse  “Many skilled expatriate nurses feel stuck in their current roles due to promotion restrictions.”- Filipino Nurse, ER Nurse* |
|  | 1. Social and Family Constraints | 1. Restricted family sponsorship | *“Being away from my spouse and children for years is emotionally draining.”- Indian Nurse, Surgical Ward  “If family visas were easier to obtain, I would be more willing to commit long-term.”-*  *Filipino Nurse, ICU Nurse* |
|  |  | 1. Adjusting to cultural norms and restrictions | *“Adjusting to cultural norms was challenging at first, but I have adapted over time.”- Pakistani Nurse, Staff Nurse  “There should be more community support programs for expatriate nurses.”- Jordanian Nurse, Nurse Manager* |
| 1. **IV: Retention Strategies (Ways to Improve Retention)** | 1. Enhancing Financial Incentives | 1. Performance-based bonuses | *“Retention bonuses for nurses who have been here for over five years would encourage long-term commitment.”- Indian Nurse, ER Nurse  “Higher salaries should reflect the cost of living and responsibilities of our job.”- South African Nurse, Charge Nurse* |
|  |  | 1. Regular salary adjustments | *“If housing and transportation allowances were increased, it would ease financial burdens.”- British Nurse, ICU Nurse  “Offering child education benefits would make long-term employment more attractive.”- Filipino Nurse, Surgical Ward* |
|  | 1. Career Development Opportunities | 1. Providing structured mentorship | *“Providing structured career paths for expatriates would make staying in Saudi Arabia more appealing.”- Jordanian Nurse, Nurse Manager  “Mandatory professional development programs should be accessible to all nurses.”- Malaysian Nurse, Staff Nurse* |
|  |  | 1. Expanding training and specialization options | *“Leadership training should be introduced to help nurses transition into higher roles.”- Pakistani Nurse, Surgical Ward  “Scholarships for higher education would motivate more nurses to stay.”- Indian Nurse, NICU Nurse* |
|  | 1. Improving Work-Life Balance | 1. Flexible scheduling | *“A better shift rotation policy would help nurses maintain a work-life balance.”- Filipino Nurse, ER Nurse  “More leave options would help prevent burnout and improve mental well-being.”- Filipino Nurse, Charge Nurse* |
|  |  | 1. More family-friendly policies | *“Providing mental health support for nurses would improve job satisfaction.”- British Nurse, Nurse Educator  “Expanding family sponsorship options would increase expatriate nurse retention.”- Indian Nurse, ICU Nurse* |
|  | 1. Strengthening Support Systems | 1. Better integration of expatriate nurses | *“Expatriate nurses should have representation in hospital leadership committees.”- Pakistani Nurse, Senior Nurse  “More cultural integration programs would help new nurses settle in faster.”- Jordanian Nurse manager* |
|  |  | 1. More inclusive decision-making processes | *“A dedicated HR team for expatriates would ensure that our concerns are addressed.”- South African Nurse, Nurse Supervisor  “Mentorship programs for new hires would improve retention and overall job satisfaction.”- Filipino Nurse, Surgical Ward* |
